# Supplementary material for: Breakdown of microbial networks links nutrient stress and reef coral disease
Source: Nat Commun. 2026 May 5;17:3821. doi: 10.1038/s41467-026-72175-4 (PMC13144614; doi:10.1038/s41467-026-72175-4)
Supplement: Supplementary file 1 — Supplementary Information [file 41467_2026_72175_MOESM1_ESM.pdf]

SUPPLEMENTARY INFORMATION

Breakdown of microbial networks links nutrient stress and reef coral disease

Raphaela Gracie, Jörg Wiedenmann, Phyllis Lam, Michael Sweet and Cecilia D'Angelo*

Supplementary Table 1 A-E: Taxonomic alignment of representative taxa of key functional groups detected in BBD lesion communities in the natural environment with taxa found in the present study. Taxa found in natural BBD communities are shaded in grey. Corresponding taxa detected in the present study down to the resolved taxonomic level are shaded in green. *Cyanobacteriota* containing taxa with nitrogen fixation potential are highlighted in orange. The "Comment" column provides further information. The Ctr, Ht and BBDI columns indicate in which sample types the taxa were identified in the present study. Ctr = control colonies, Ht = Visually healthy tissue on BBD diseased colonies, BBD = Microbial mats from BBD lesions

A — ***Cyanobacteriota* (mat / habitat forming, creation of microenvironment)**

Phylum	Class	Order	Family	Genus	Ctr	Ht	BBD	Comment
Cyanobacteriota	*Cyanobacteriia / Cyanphyceae*	*Cyanobacteriales*	*Desertifilaceae*	*Oscillatoria*<br><br>Some species fix nitrogen[1, 2, 3, 4, 5]				BBD in Bahamas, St Croix, Philippines, and Florida Keys[6, 7]<br><br>Pre-BBD/BBD lesions Great Barrier Reef[8]
Cyanobacteriota	*Cyanobacteriia / Cyanphyceae*	*Cyanobacteriales*	*Desertifilaceae*	*Oscillatoria*		x		
				Incertae Sedis_507	x	x	x	
Cyanobacteriota	*Cyanobacteriia / Cyanphyceae*	*Cyanobacteriales*	*Desertifilaceae*<br><br>Some taxa fix nitrogen[4]	*Hormoscilla*				Belize BBD[7, 9]<br><br>Related to *Roseofilum*
Cyanobacteriota	*Cyanobacteriia / Cyanphyceae*	*Cyanobacteriales*	*Desertifilaceae*	*Hormoscilla*	x	x	x	BBD lesions, ASV325 top BLAST match (99.75%) with Uncultured bacterium clone from BBD lesions in *Favia* sp. corals from Eilat.
Cyanobacteriota	*Cyanobacteriia / Cyanphyceae*	*Cyanobacteriales*	*Desertifilaceae*<br><br>Some taxa fix nitrogen[4]	*Roseofilum*<br><br>Some species fix nitrogen[10]				BBD in *Sidastrea* from Belize/Florida[7]<br><br>*Roseofilum/Phormidium corallyticuum* is not always present in BBD mats. Instead, the dominant cyanobacteria in the mat are context dependent[11, 12].

Cyanobacteriota	*Cyanobacteriia / Cyanphyceae*	*Cyanobacteriales*	*Desertifilaceae*	Not detected	x	x	x	
Cyanobacteriota	*Cyanobacteriia / Cyanphyceae*	*Cyanobacteriales*	*Geitlerinemaceae*	*Geitlerinema*<br><br>Some species fix nitrogen[13]				BBD in Bahamas and Florida Keys[6, 7]
Cyanobacteriota	*Cyanobacteriia / Cyanphyceae*	*Cyanobacteriales*	*Geitlerinemaceae*	*Geitlerinema*	x			
Cyanobacteriota	*Sericytochromatia*	*Leptolyngbyales*	*Leptolyngbyaceae*<br>Some taxa fix nitrogen[4]	*Leptolyngbya*<br><br>Some species fix nitrogen[4]				BBD in Bahamas, Philippines, St. Croix and Florida Keys[6, 7]
Cyanobacteriota	*Sericytochromatia*	*Leptolyngbyales*	*Leptolyngbyaceae*	*Leptolyngbya*		x		
Cyanobacteriota	*Sericytochromatia*	*Leptolyngbyales*	*Leptolyngbyaceae*	*Incertae Sedis_514*	x	x		
Cyanobacteriota	*Sericytochromatia_2920*	*Phormidesmiales*	*Phormidesmiaceae*	*Phormidium*<br><br>Some species fix nitrogen[3]				BBD in Bahamas and Florida Keys[7]
Cyanobacteriota	*Sericytochromatia_2920*	*Phormidesmiales*	*Phormidesmiaceae*	*Phormidium*	x	x	x	BBD diseased colony, ASV6242 has a top BLAST match (100%) with Filamentous cyanobacterium FLK9 from BBD lesions taken from corals in the Northern Florida Keys[14].<br><br>BBD lesion, ASV14894 has a 98.01% sequence match with Cyanobacterium sp. BBD-AO-Green from black band disease from corals (Buerger et al., unpublished, see GenBank KU720413.1).
Cyanobacteriota	*Cyanobacteriia / Cyanphyceae*	*Cyanobacteriales*<br><br>Some taxa fix nitrogen[15, 16]	*Paraspirulinaceae*	*Spirulina*				BBD - Microscopic ID[14, 17, 18]
Cyanobacteriota	*Cyanobacteriia / Cyanphyceae*	*Cyanobacteriales*	*Paraspirulinaceae*	*Spirulina*	x	x	x	BBD lesions, ASV43 has a 99.01% sequence match with Cyanobacterium BBT from BBD bacterial mats (Frias-Lopez et al., unpublished, see GenBank AY515014.1).<br><br>BBD lesions, ASV182 has a 99.26% ssequence match with Uncultured bacterium clone CD02013E12 from a BD mat on a Gorgonia ventalina colony from Curacao[19].

			Xenococcaceae	*unclassified_Xenococcaceae*	x	x	x	
			Xenococcaceae	*Pleurocapsa PCC-7319*	x	x	x	
			Xenococcaceae	*Chroococcidiopsis PCC-6712*	x	x	x	
			Nostocaceae	*Rivularia* PCC-7116<br>Some species fix nitrogen[16]	*x*			
			Nostocaceae	*Mastigocoleus BC008*<br>Some species fix nitrogen[15]	x	x		
			Cyanobacteriaceae	*Geminobacterium*	x	x	x	
			Cyanobacteriaceae	*Symphothece PCC-7002*	x			
			Cyanobacteriaceae	*Merismopedia AICB1015*		x		
			Cyanobacteriaceae	*Annamia HOs24*	x	x		
			Coleofasciculaceae	*Caldora VP642b*	x			
Cyanobacteriota	*Cyanobacteriia / Cyanphyceae*	*Eurycoccales*	*Eurycoccales Incertae Sedis*	*Synechococcus*<br>Some species fix nitrogen[5, 20, 21]				BBD in Great Barrier Reef *Montipora*[8]
Cyanobacteriota	*Cyanobacteriia / Cyanphyceae*	*Eurycoccales*	*Eurycoccales Incertae Sedis*	*Synechococcus*	x	x	x	
Cyanobacteriota	*Sericytochromatia_424*	*Limnotrichales_2*	*Limnotrichaceae_2*	*Limnothrix*				Pre-BBD lesions Great Barrier Reef[8]
Cyanobacteriota	*Sericytochromatia_424*	*Limnotrichales_2*	*Limnotrichaceae_2*	*Limnothrix*	x	x		
Cyanobacteriota	*Sericytochromatia_424*	*Pseudanabaenales_2*	*Pseudanabaenaceae_2*	*Pseudanabaena*<br>Some species fix nitrogen[3]				BBD Red Sea[22]
Cyanobacteriota	*Sericytochromatia_424*	*Pseudanabaenales_2*	*Pseudanabaenaceae_2*	*Pseudanabaena*	x	x		
		Obscuribacterales	*Obscuribacteraceae*	*Incertae Sedis_528*	x	x	x	
		Phormidesmiales	*Nodosilineaceae*	*MBIC10086*	x	x	x	BBD lesions, ASV153 found in BBD had a top BLAST match (100%) with Uncultured bacterium clone SGUS386 from White Plague Disease associated communities in *Montastraea faveolata* from the Caribbean[23].

		Phormidesmiales	*Nodosilineaceae*	*unclassified_Nodosilineaceae*		x	x	BBD diseased colony, ASV10931 had a 100% BLAST sequence match with Uncultured cyanobacterium clone Feb_09_5S.7 from BBD consortia from Favia sp. in the Red Sea[24].
		Phormidesmiales	*Phormidesmiaceae*	*Phormidesmis*	x	x	x	
		Phormidesmiales	*Phormidesmiaceae*	*Acrophormium*	x	x	x	
		Thermosynechococcales	*Acaryochloridaceae*	*Acaryochloris*	x	x	x	
Cyanobacteriota	*Sericytochromatia_6194*	*Synechococcales*<br>Some taxa fix nitrogen[3]	*Synechococcales Incertae Sedis*	*Schizothrix*<br>Some species fix nitrogen[25]				Microscopic identification[14, 26]
Cyanobacteriota	*Sericytochromatia_6194*	*Synechococcales*	*Synechococcales Incertae Sedis*	*Schizothrix*	x	x	x	BBD diseased colony, ASV4183 99.75% BLAST match with Uncultured cyanobacterium clone MD3.8 from diseased colonies of *Montastrea faveolata* from the Caribbean (Johnson et al. unpublished, see GenBank FJ425596.1).
			Cyanobiaceae	*Cyanobium PCC-6307*	x	x	x	BBD lesions, ASV2092 99.5% sequence match with Uncultured cyanobacterium clone FRSSCT_14f from BBD coral tissue of *Siderastrea siderea* from St. Croix[27].
	Vampirivibrionia	*Caenarcaniphilales*	*Incertae Sedis_235*	*Incertae Sedis_526*	x	x	x	
	Vampirivibrionia	*Gastranaerophilales*	*Gastranaerophilaceae*	*Incertae Sedis_527*	x			

**B**			**Sulfate-reducing bacteria (Production of toxic sulfide)**					
**Phylum**	**Class**	**Order**	**Family**	**Genus**	**Ctr**	**Ht**	**BBD**	**Comment**
Thermo desulfobacteriota	***Desulfobacteria***	***Desulfobacterales***	***Desulfobacteraceae***	***Desulfobacter***				**BBD in corals in Curacao[19]**<br><br>**Increased in bleached corals from South China Sea[28]**
Thermo desulfobacteriota	***Desulfobacteria***	***Desulfobacterales***	***Desulfobacteraceae***	***Desulfobacter***	**x**	**x**	**x**	
				unclassified_Desulfobacteraceae	**x**	**x**	**x**	
			Desulfosarcinaceae	***Desulfosarcina***			**x**	
			Incertae Sedis_481	***Incertae Sedis_999***		**x**		
			Desulfolunaceae	***unclassified_Desulfolunaceae***		**x**		
			Desulfosarcinaceae	***Incertae Sedis_998***			**x**	**BBD lesions**, ASV18520 has a 100% BLAST sequence match with Uncultured delta proteobacterium clone D10 from anaerobic marine sediments (Koachling et al., unpublished, see GenBank GQ249552).
Thermodesulfobacteriota	***Desulfovibrionia***	***Desulfovibrionales***	***Desulfovibrionaceae***	***Halodesulfovibrio***				**BBD from the Red Sea, Israel[31]**
Thermodesulfobacteriota	*Desulfovibrionia*	*Desulfovibrionales*	*Desulfovibrionaceae*	*Halodesulfovibrio*	x	x	x	**BBD lesions,** ASV5642 has a 99.77% sequence match with Uncultured bacterium clone BBD4-907-79 from BBD mat from a *Favia* sp. coral from the Red Sea, Israel[31].<br><br>**BBD lesions,** ASV5642 has a 99.77% sequence match with Uncultured bacterium clone BBD-Aug08-4BB-79 from BBD mat from a *Favia* sp. coral from the Red Sea, Israel (Arotsker et al., unpublished, see GenBank GU472053.1).<br><br>**BBD lesions,** ASV5642 also has a 99.77% sequence match with Delta proteobacterium BBD 3-1 from BBD from *Diplora clivosa* from Florida (Viehman et al., unpublished, see GenBank AY750147.1).<br><br>**BBD lesions,** ASV1431 has a 100% BLAST sequence match with Uncultured *Desulfovibrio* sp. clone MD3.10 found in diseased colonies of the Caribbean coral

								Montastrea faveolata (Johnson et al., unpublished, see GenBank FJ425598.1).<br><br>**BBD infected coral,** ASV4061 has a 100% sequence match with Uncultured bacterium clone BBD-Aug08-4BB-106 found in a BBD affected *Favia* sp. coral from Eilat (Arotsker et al., unpublished, see GenBank GU472076.1).<br><br>**BBD infected coral,** ASV4061 also has a 100% sequence match with Delta proteobacterium BBD 11-2 from BBD lesions from a *Siderastrea sidereal* coral from Dominica (Viehman et al., unpublished, see GenBank AY750148).<br><br>**BBD lesions,** ASV9131 has a 99.77% sequence match with Uncultured Desulfovibrio sp. clone MD3.10 from diseased colonies of *Montastraea faveolata* from the Caribbean (Johnson et al. unpublished, see GenBank FJ425598.1).<br><br>**BBD lesions,** ASV9131 has a 99.77% sequence match with Uncultured bacterium clone SGUS1499 from diseased corals of *Montastraea faveolata*[23].<br><br>**BBD infected colony**, ASV4061 has a 100% sequence match with Uncultured bacterium clone BBD-Aug08-4BB-106 from BBD affected *Favia* sp. corals from Eilat, Israel (Arotsker et al., unpublished, see GenBank GU472076.1).<br><br>**BBD infected colony**, ASV4061 has a 100% sequence match with Delta proteobacterium BBD 11-2 from BBD of *Siderastrea siderea* from Dominica (Viehman et al., unpublished, see GenBank AY750148.1).<br><br>**BBD infected colony**, ASV5177 has a 99.53% sequence match with Uncultured bacterium clone BBD4-907-79 from BBD mats from *Favia* sp. from the Red Sea, Israel[31].
Thermodesulfobacteriota	***Desulfovibrionia***	***Desulfovibrionales***	***Desulfovibrionaceae***	***Desulfovibrio***				**BBD in Red Sea**[22, 29]

								**BBD on multiple Caribbean coral species[30]**
Thermodesulfobacteriota	*Desulfovibrionia*	*Desulfovibrionales*	*Desulfovibrionaceae*	*Pseudodesulfovibrio*	x	x	x	
				Maridesulfovibrio	x			
				unclassified_Desulfovibrionaceae	x			
				Incertae Sedis_1006	x			
				Incertae Sedis_876	x	x	x	
Thermodesulfobacteriota	***Desulfobulbia***	***Desulfobulbales***	***Desulfobulbaceae***	***Desulfobulbus***				**Increased in bleached corals from South China Sea[28]**
Thermodesulfobacteriota	*Desulfobulbia*	*Desulfobulbales*	*Desulfobulbaceae*	*unclassified_Desulfobulbaceae*	x		x	

C	Sulfide-oxidizing bacteria (Maintaining the sulphur metabolism)							
**Phylum**	**Class**	**Order**	**Family**	**Genus**	**Ctr**	**Ht**	**BBD**	**Comment**
Pseudomonadota	***Gammaproteobacteria***	***Beggiatoales***	***Beggiatoaceae***	***Beggiatoa***				**BBD in the Red Sea**[22]<br><br>**BBD in the wider Caribbean**[7, 32]<br><br>**BBD in Bermuda**[18]
Pseudomonadota	*Gammaproteobacteria*	*Beggiatoales*	*Beggiatoaceae*	*Unclassified Beggiatoaceae*	x	x	x	**BBD lesions,** Beggiatoaceae are Nitrate-dependent sulfide oxidizing bacteria[33]
Pseudomonadota	*Gammaproteobacteria*	*Beggiatoales*	*Beggiatoaceae*	*Thioflexithrix*	x	x	x	**BBD lesions,** Sulfide-oxidising genus. ASV478 has a 100% sequence match with *Beggiatoa* sp. 35Flor from BBD lesions in a Scleractinian coral from the Florida Keys[34].<br>Filamentous, sulfide-oxidizing bacterium[35].
Campylobacterota	***Campylobacteria***	***Campylobacterales***	***Arcobacteraceae***	***Arcobacter***				**BBD in the Red Sea**[22]<br><br>**BBD in corals in Curacao**[19]
Campylobacterota	*Campylobacteria*	*Campylobacterales*	*Arcobacteraceae*	*Arcobacter*	x	x	x	**BBD lesions**, ASV31 has a 100% sequence match with Uncultured bacterium clone CD02013E10 found in BBD mats of *Gorgonia ventalina*[19]<br><br>**BBD lesions**, ASV18 has a 100% sequence match with Uncultured bacterium clone SHFG542 from diseased tissue (White Plague Disease) of *Montastraea faveolate* from the Caribbean[23].
Campylobacterota	***Campylobacteria***	***Campylobacterales***	***Sulfurospirillaceae***	**Sulfurospirillum**				**BBD in the Red Sea**[22]
Campylobacterota	Campylobacteria	Campylobacterales	Sulfurospirillaceae	*Sulfurospirillum*	x			
Campylobacterota	Campylobacteria	Campylobacterales	Sulfurimonadaceae	*Sulfurimonas*	x	x		Sulfide-oxidising genus<br><br>*Sulfurimonas* can reduce nitrate to nitrite or N_2, enabling anaerobic growth via denitrification[36].

D			Heterotrophic & opportunistic bacteria					
Phylum	Class	Order	Family	Genus	Ctr	Ht	BBD	Comment
Pseudomonadota	***Gammaproteobacteria***	***Enterobacterales***	***Vibrionaceae***	***Vibrio***				**BBD-infected corals in the Red Sea[29]**<br><br>**Vibrio isolated from BBD bands, culture and proteolytic activity tested[37]**<br><br>**Vibrio isolated from BBD bands in the wider Caribbean[38]**
Pseudomonadota	*Gammaproteobacteria*	*Enterobacterales*	*Vibrionaceae*	*Vibrio*	x	x	x	**BBD lesions,** ASV1406 has a 100% sequence match with Vibrio sp. BD6B from the sulphurus layer of a diseased *Montipora aequituberculata* skeleton with White Syndrome[39].
				Unclassified Vibrionaceae	x	x	x	**BBD lesions,** ASV45 has a 100% sequence match with *Vibrio Pelagius* strain 5100744_CD3_3 from diseased corals with White Syndrome from Malaysia (Akmal et al., unpublished, see GenBank PP980502.1) and a 100% sequence match with *Vibrio* sp. 4H1 from a *Montastraea faveolata* coral with Yellow Band Disease from Puerto Rico (Cunning et al., unpublished, see GenBank EU517650.1).
				Photobacterium	x	x	x	
				Grimontia	x			
				Salinivibrio	x		x	
				Aliivibrio	*x*			
				Enterovibrio	x	x		
				Catenococcus_2	x		x	
Pseudomonadota	***Gammaproteobacteria***	***Enterobacterales***	***Pseudoalteromonadaceae***	***Pseudoalteromonas***				**Isolates from BBD bands in the wider Caribbean[40]**<br><br>**Identified in BBD bands from Indonesia[41, 42]**

Pseudomonadota	*Gammaproteobacteria*	*Enterobacterales*	*Pseudoalteromonadaceae*	*Pseudoalteromonas*	x	x	x	**BBD lesions,** ASV2320 has a 100% sequence match with Pseudoaltermonas sp. Pad1.13 from diseased gorgonian *Pseudopterogorgia americana* (Vizcaino et al., unpublished, see GenBank GQ406581.1).
				Algicola	x	x	x	**BBD lesions,** ASV48 has a 100% sequence match on BLAST with Uncultured bacterium clone RSAE3C39 found in the *Porites* White Patch Syndrome-infected tissue of corals from South Africa (Sere et al., unpublished, see GenBank KF180023.1).<br><br>**BBD lesions,** ASV1177 has a 99.77% sequence match with Uncultured bacterium clone Thai19_H05 from White Plague Disease infected coral *Pavona duerdeni* [43].
				unclassified_Pseudoalteromonadaceae	x	x	x	
				Psychrosphaera	x		x	
				Incertae Sedis_894	x	x		
Pseudomonadota	***Gammaproteobacteria***	***Enterobacterales***	***Alteromonadaceae***	***Alteromonas***				**BBD mats in the Red Sea[22]**<br><br>**BBD mats in the wider Caribbean[44]**<br><br>**BBD mats in the GBR[8]**
Pseudomonadota	*Gammaproteobacteria*	*Enterobacterales*	*Alteromonadaceae*	*Alteromonas*	x	x	x	
				Incertae Sedis_880	x	x	x	
				unclassified_Alteromonadaceae_2	x	x	x	
				Aestuariibacter		x	x	
				Marisediminitalea	x			
				Planctobacterium	x	x	x	
				Glaciecola	x	x		
				Neptunicella	x			
				Lacimicrobium	x			
				Aliiglaciecola	x	x		
				Paraglaciecola	x			

Pseudomonadota	***Alphaproteobacteria***	***Rhodobacterales***	***Paracoccaceae***	***Ruegeria***				**BBD mats in the wider Caribbean**[7, 11, 32]<br><br>**BBD mats in Okinawa, Japan**[45]
Pseudomonadota	*Alphaproteobacteria*	*Rhodobacterales*	*Paracoccaceae*	*Ruegeria*	x	x	x	**BBD lesions,** ASV17 has a 100% sequence BLAST match with Uncultured bacterium clone BBD-Aug08-3BB-31 from BBD affected *Favia* sp. corals in Eilat, Israel (Arotsker et al., unpublished, see GenBank GU472124.1).<br><br>**BBD lesions,** ASV138 has a 99.75% sequence match with Uncultured bacterium clone BBDS16S-15 from BBD mats from *Favites* sp. from the Red Sea[29].<br><br>**BBD lesions**, ASV752 has a 100% sequence match with Uncultured bacterium clone SHFG523 from diseased tissue of White Plague Disease infected *Montastraea faveolata* from the Caribbean[23].
				unclassified_Paracoccaceae	x	x	x	**BBD lesions**, ASV9 has a 100% sequence match with Uncultured alpha proteobacterium clone BBD_217_23 from BBD diseased coral tissue of *Sidestrea siderea* from Florida[46].<br><br>**BBD lesions**, ASV15 has a 100% sequence match with Uncultured bacterium clone SHFG549 from diseased tissue of *Montastraea faveolata* infected with White Plague Disease from the Caribbean[23].
				Shimia	x	x	x	**BBD lesions,** ASV64 has a 100% sequence match with Uncultured bacterium clone BBD-Aug08-1BB-18 from BBD affected coral *Favia* sp. in Eilat, Israel (Arotsker et al., unpublished, see GenBank GU472113.1).
				Cognatishimia	x	x	x	**BBD lesions,** ASV76 has a 99.25% sequence match with Uncultured alpha proteobacterium clone BBD_HS216b_07 found in BBD coral tissues of *Siderastrea siderea* in the Bahamas (Richardson et al., unpublished, see GenBank DQ644016.1).
				Dinoroseobacter	x	x	x	
				Roseobacter clade CHAB-I-5 lineage	x	x	x	**BBD lesions**, ASV300 has a 100% sequence match with Uncultured bacterium clone SGUS600 found in *Montasraea*

								faveolata study looking at White Plaque Disease[23].
				Pikeienuella	x	x	x	
				Marivita	x	x	x	
				Silicimonas	x	x	x	**BBD lesions,** ASV390 has a 99.75% sequence match with Uncultured alpha proteobacterium B7-20G from *Porites lobata* with BBD from the Great Barrier Reef (Cooney et al., unpublished, see GenBank AY148320.1).
				Actibacterium	x	x	x	**BBD lesions,** ASV808 has a 99.75% sequence match with Uncultured bacterium clone SGUS1403 found in *Montasraea faveolata* study looking at White Plaque Disease[23].
				Limibaculum	x	x	x	
				Tropicibacter	x		x	**BBD lesions,** ASV1316 has a 99.5% sequence match with Uncultured bacterium clone BBDS16S from BBD mats from *Favites* sp. from the Red Sea[29].
				Roseovarius	x	x	x	
				Sulfitobacter	x			Found in early stages of BBD infection in corals[8].
				Rubrimonas	x	x		
				Tropicibacter	x		x	**BBD lesions,** ASV1316 has a 99.5% sequence match with Uncultured bacterium clone BBDS16S-10 from BBD mats from *Favites* sp. in the Red Sea [29].
				Yoonia	x	x	x	**BBD lesions,** ASV2636 has a 99.25% sequence match with Uncultured alpha proteobacterium clone CL18-G05 from a cyanobacterial patch stage of BBD development in *Montipora hispida* from the Great Barrier Reef[8].
				Tateyamaria	x		x	
				Tranquillimonas	x	x	x	
				Muriiphilus		x		
				Incertae Sedis_811	x	x	x	**BBD lesions,** ASV10716 is a 100% sequence match with Uncultured bacterium clone BB31NT16S-9 found in *Favites* sp.

								mucus adjacent to BBD mat in the Red Sea[29].
				Allosediminivita	x	x		
				Lentibacter	x	x		
				Limimaricola	x			
				Planktomarina	x			
				Maribius	x			
				Loktanella	x			
				Chachezhania		x		
Pseudomonadota	***Gammaproteobacteria***	***Pseudomonadales***	***Marinobacteraceae***	***Marinobacter***				**BBD mats in the Bahamas[47]**<br><br>**BBD mats in the wider Caribbean[48]**
Pseudomonadota	*Gammaproteobacteria*	*Pseudomonadales*	*Marinobacteraceae*	*Marinobacter*	x	x		
Bacteroidota	***Bacteroidia***	***Flavobacteriales***	***Flavobacteriaceae***	***Flavobacterium***				**BBD mats in the wider Caribbean[11, 32]**
Bacteroidota	*Bacteroidia*	*Flavobacteriales*	*Flavobacteriaceae*	*Flavobacterium*	x	x		
				Winogradskyella	x	x	x	**BBD lesions,** ASV335 has a 100% sequence match with Uncultured *Winogradskyella* sp. clone MD3.56 in diseased Caribbrean *coral Montastrea faveolate* (Johnson et al., unpublished, see GenBankFJ425644.1).
				Tenacibaculum	x	x	x	
				Pseudotenacibaculum	x	x	x	

E			Recurrent secondary genera detected in BBD sequencing reports					
Phylum	Class	Order	Family	Genus	Ctr	Ht	BBD	Comment
Spirochaetota	***Sericytochromatia_6043***	***Spirochaetales***	***Spirochaetaceae***	***Spirochaeta***				**Red Sea BBD**[22]<br><br>**Caribbean BBD**[32]
Spirochaetota	*Sericytochromatia_6043*	*Spirochaetales*	*Spirochaetaceae*	*Spirochaeta*	x	x	x	**BBD lesions,** ASV8588 has a 100% sequence match with Uncultured Spirochaetes bacterium clone 1HP1-B8 from a ***Turbinaria** mesenterina* colony affected by White Syndrome in Australia (Godwin et al., unpublished, see GenBank EU780318.1).
				Sediminispirochaeta	x	x	x	
				Oceanispirochaeta	x		x	
				Thiospirochaeta	x			
Bacillota	***Sericytochromatia_2834***	***Peptostreptococcales-Tissierellales***	***Fusibacteraceae***	***Fusibacter***				**Red Sea BBD**[22]<br><br>**Caribbean BBD**[32, 44, 48]<br><br>**BBD wider Caribbean**[49]
Bacillota	*Sericytochromatia_2834*	*Peptostreptococcales-Tissierellales*	*Fusibacteraceae*	*Fusibacter*	x	x	x	**BBD lesions,** ASV25 has a 100% sequence match with Uncultured bacterium clone BBD4-907-2 from a BBD mat from *Favia* sp. in Eilat, Israel[31].<br><br>**BBD lesions**, ASV63 has a 99.75% sequence match with Uncultured bacterium clone BBD4-907-2 from BBD mats on infected *Favia* sp. corals from the Red Sea[31].<br><br>**BBD lesions**, ASV63 has a 99.75% sequence match with Uncultured Firmicutes bacterium clone WA_20bf from BBD infected coral tissues from *Siderastrea sidereal* corals from the wider Caribbean [32].<br><br>**BBD lesions,** ASV99 has a 100% sequence match with Uncultured bacterium clone CD02002D01 from BBD mat from *Colpophyllia natans* from Curacao[19].<br><br>**BBD lesions**, ASV374 has a 99.75% sequence match with Uncultured bacterium

								clone BB3S16S-1 from BBD mat from *Favites* sp. corals from the Red Sea[29].
Bacillota	***Clostridia***	***Clostridiales***	***Clostridiaceae***	***Clostridium***				**Red Sea BBD[22]**<br>**GBR BBD[8]**<br>**Caribbean BBD[44]**
Bacillota	*Clostridia*	*Clostridiales*	*Clostridiaceae*	*Clostridium*	x			
				unclassified_Clostridiaceae	x			
				Oceanirhabdus	x			
				Incertae Sedis_220	x			

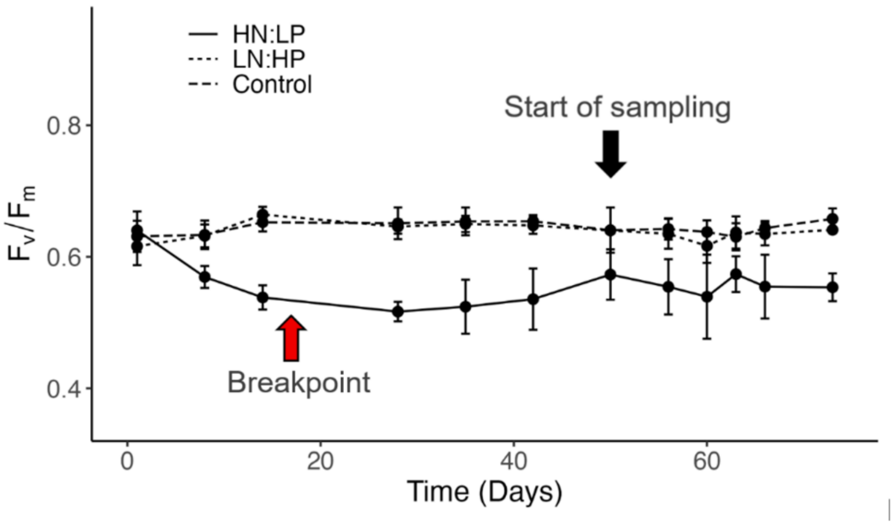

Supplementary Figure 1: Effect of seawater N:P stoichiometry on maximum quantum yield of Photosystem II (Fv/Fm). Mean ± standard deviation of Fv/Fm values are shown for T. reniformis under Control, HN:LP and LN:HP conditions (n = 6 replicate colonies per nutrient condition). Type III ANOVA with Satterthwaite's method indicated significant main effect of nutrient treatment ($F(2, 75.271) = 20.615$, $p < 0.001$) when comparing HN:LP conditions with either Control or LN:HP conditions. No significant differences were detected between Control or LN:HP conditions. Segmented regression analysis of the HN:LP conditions identified a significant breakpoint at day 16.7 +/- 2.3 SE (red arrow), transitioning from a rapid decline (slope = -0.0079, $p < 0.001$) to a stable state. No significant declines or breakpoints were identified in Fv/Fm time courses of the Control and the LNHP groups. The start of sampling at day 50 (black arrow) took place after the corals were fully acclimatised to the respective nutrient conditions.

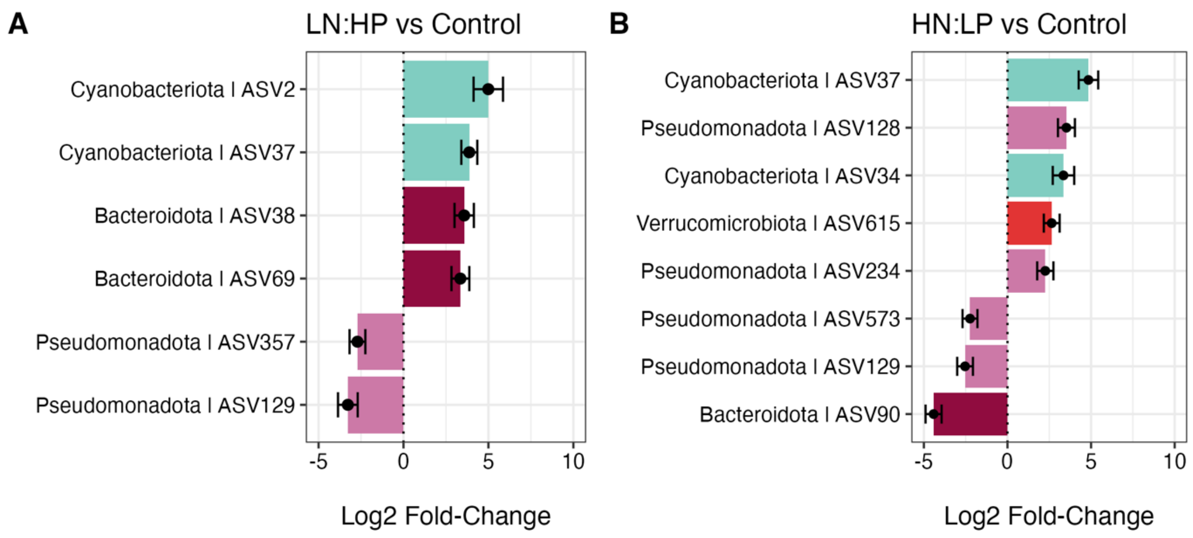

Supplementary Figure 2: Coral-associated bacterial taxa with a significant change in relative abundance under skewed seawater N:P stoichiometry for (**A**) LN:HP and (**B**) HN:LP relative to control conditions (total n = 32 samples). Data show mean effect size (log fold change) and 95% confidence interval bars (two-sided; FDR corrected) derived from the ANCOM-BC2 model of pooled BBD and healthy tissue-derived sequences at the Amplicon Sequence Variant (ASV) level. Only bacterial taxa of an effect size with an adjusted $p < 0.05$ are shown. Since the analysis returns only sequences that are enriched compared to the control, the displayed sequences are present in the control samples as well, yet at lower relative abundance. ASV37, enriched under both, LN:HP and HN:LP, is closely related to sequences from BBD communities reported in the literature (Suppl. Fig. 4). ASV2 has 100% match with HM768653, a sequence from a BBD community of a seafan[19].

A

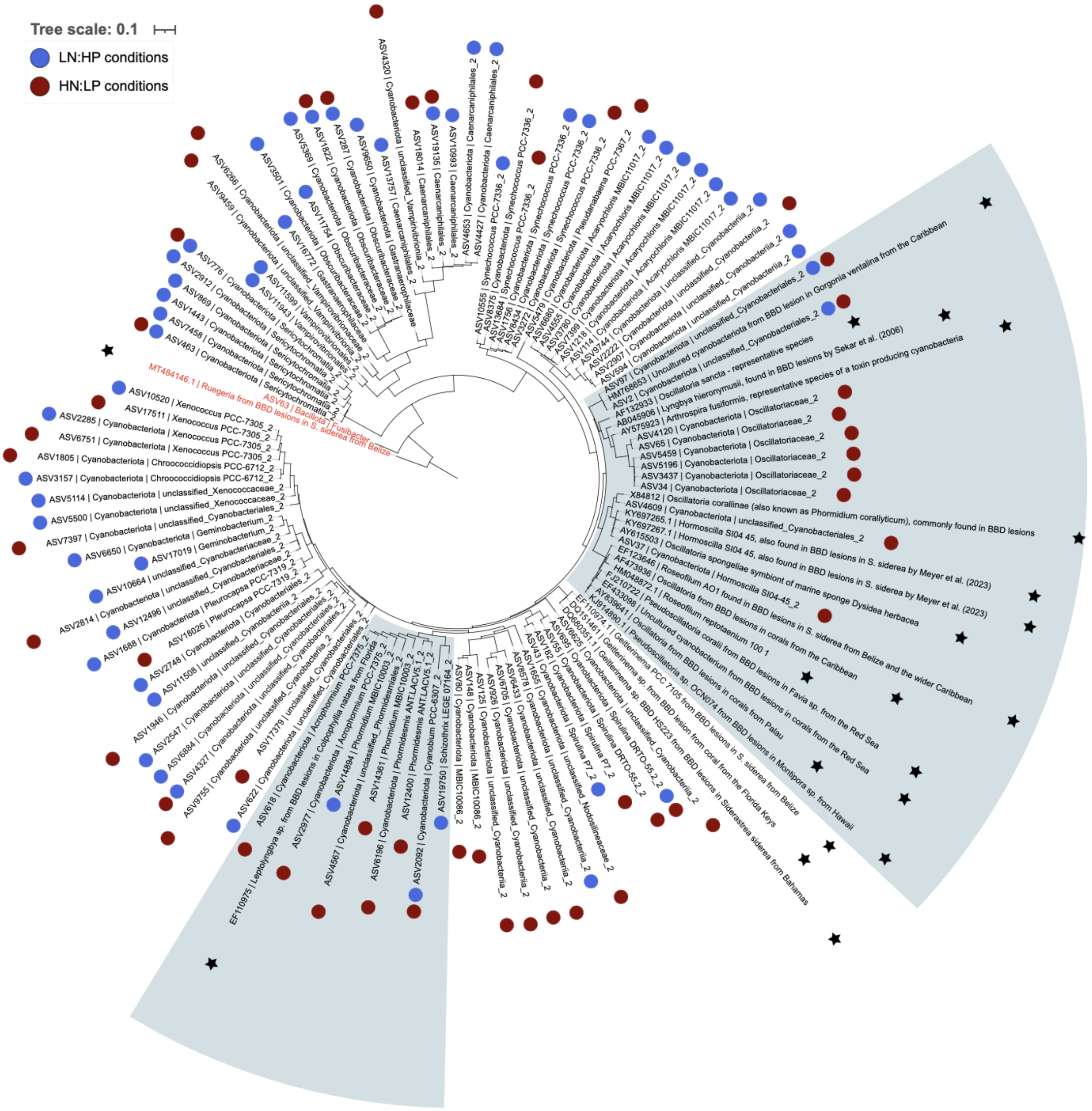

B

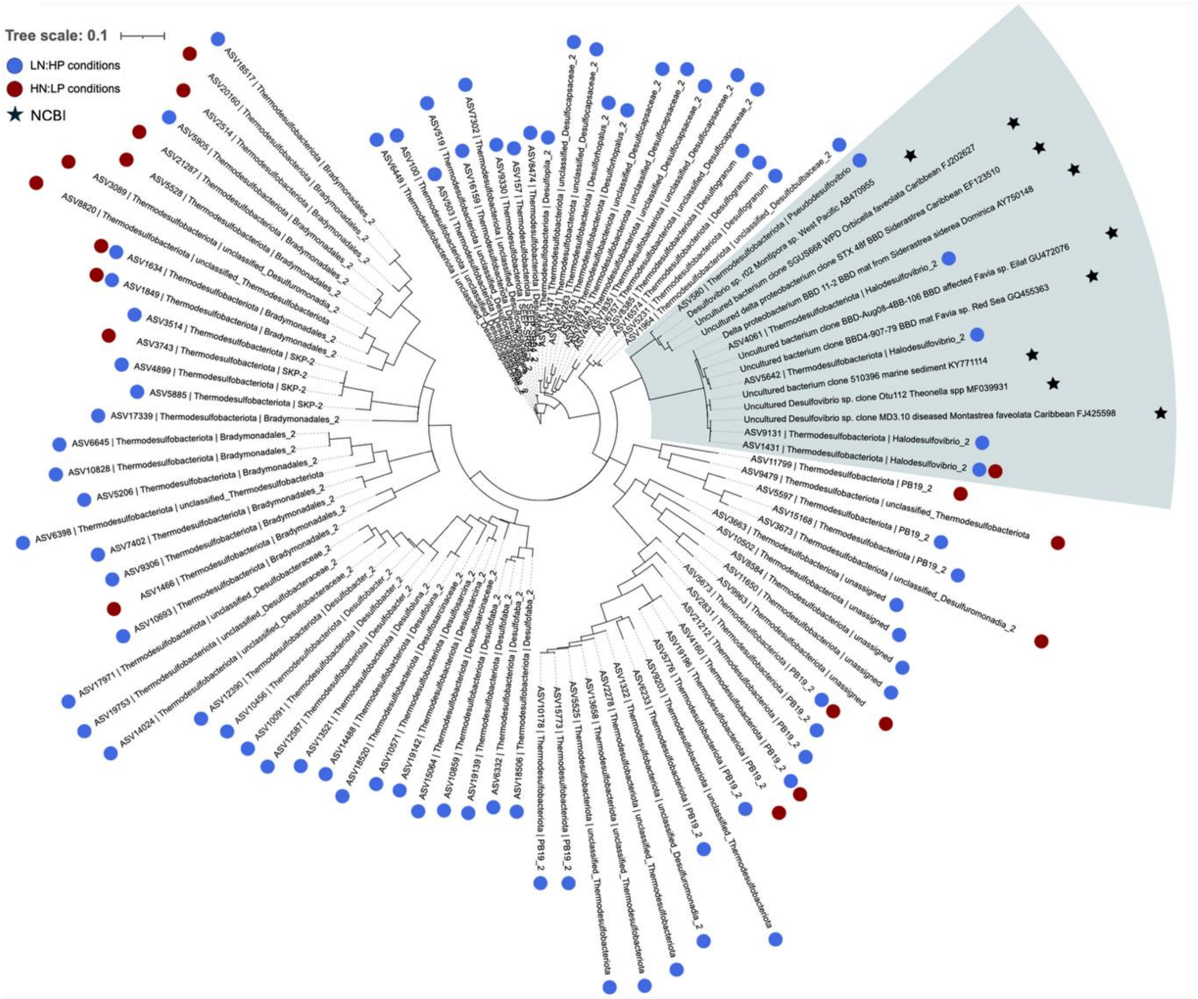

Supplementary Figure 3: Phylogeny of 16S rRNA amplicon sequences from (A) *Cyanobacteriota* and (B) *Thermodesulfobacterota* found in disease lesion bacterial mats (n = 4 disease lesion samples per nutrient condition). Hypothetical phylogenetic tree showing the relationship between 16S rRNA gene sequences identified in bacterial mats associated with tissue loss in *T. reniformis* and 16S rRNA gene sequences obtained from NCBI. A Maximum Likelihood phylogenetic tree of partial 16S rRNA gene sequences was constructed using IQ-TREE, with branch support estimated by 1,000 ultrafast bootstrap replicates. Spheres indicate the nutrient conditions from which each sequence was derived. Sequences obtained from NCBI for comparison contain their respective accession number and they are highlighted by an asterisk. Clusters (shade in grey) highlight groups containing closely related sequences identified in the BBD lesions from this study and NCBI-downloaded sequences obtained from BBD samples collected in distinct reef locations.

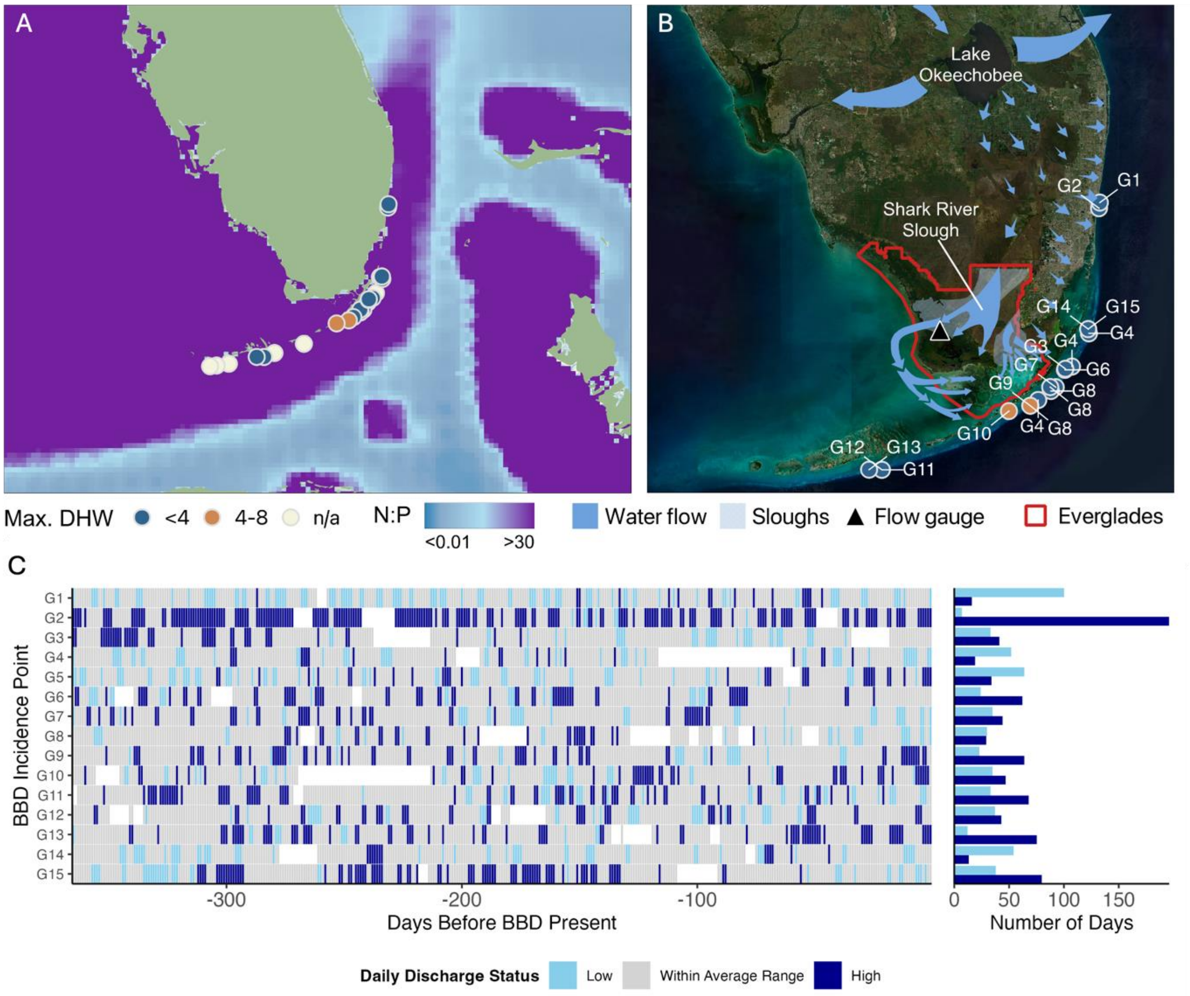

Supplementary Fig. 4: Temperature history and nutrient environment of black band disease locations along the southeast coast of Florida. A Black band incidences and prevailing N:P stoichiometric conditions. **B** Flow of water through southern Florida and the Everglades into the Florida Keys. Spheres represent incidences of black band disease where DHW data were available. Sites are grouped ('G') by the time that disease was reported (e.g. disease was reported at the same time at all sites in group 'G4'). **C** Daily mean discharge (tidally filtered) anomalies within the previous 365 days before reported BBD events. Data from the USGS flow gauge situated in Shark River Slough, Florida. Water flow directions adapted from "Progress Toward Restoring the Everglades: The Eighth Biennial Review – 2020"[50].

Supplementary References

1. Carpenter E, Price C. Marine Oscillatoria (Trichodesmium): .Explanation: Aerobic Nitrogen Fixation Without Heterocysts. *Science* **191**, 1278–1280 (1976).

2. Gallon J, Hashem M, Chaplin A. Nitrogen fixation by OsciZZatoria spp. under autotrophic and photoheterotrophic conditions *Journal of General Microbiology* **137**, 31–39 (1991).

3. Bergman B, Gallon J, Rai A, Stal L. N2-fixation by non-heterocystous cyanobacteria. *FEMS Microbiology Reviews* **19**, 139–185 (1997).

4. Stal LJ. Nitrogen Fixation in Cyanobacteria. In: *Encyclopedia of Life Sciences*) (2015).

5. Fay P. Oxygen Relations of Nitrogen Fixation in Cyanobacteria. *Microbiological Reviews* **56**, 340–373 (1992).

6. Myers JL, Sekar R, Richardson LL. Molecular detection and ecological significance of the cyanobacterial genera Geitlerinema and Leptolyngbya in black band disease of corals. *Appl Environ Microbiol* **73**, 5173–5182 (2007).

7. Meyer JL*, et al.* Cryptic Diversity of Black Band Disease Cyanobacteria in Siderastrea siderea Corals Revealed by Chemical Ecology and Comparative Genome-Resolved Metagenomics. *Mar Drugs* **21**, (2023).

8. Sato Y, Willis BL, Bourne DG. Successional changes in bacterial communities during the development of black band disease on the reef coral, Montipora hispida. *ISME J* **4**, 203–214 (2010).

9. Schorn MA*, et al.* Comparative Genomics of Cyanobacterial Symbionts Reveals Distinct, Specialized Metabolism in Tropical Dysideidae Sponges. *mBio* **10**, (2019).

10. Meyer JL, Paul VJ, Raymundo LJ, Teplitski M. Comparative Metagenomics of the Polymicrobial Black Band Disease of Corals. *Front Microbiol* **8**, 618 (2017).

11. Cooney RP, Pantos O, Le Tissier MD, Barer MR, O'Donnell AG, Bythell JC. Characterization of the bacterial consortium associated with black band disease in coral using molecular microbiological techniques. *Environ Microbiol* **4**, 401–413 (2002).

12. Frias-Lopez J, Bonheyo GT, Jin Q, Fouke BW. Cyanobacteria associated with coral black band disease in Caribbean and Indo-Pacific Reefs. *Appl Environ Microbiol* **69**, 2409–2413 (2003).

13. Den Uyl PA, Richardson LL, Jain S, Dick GJ. Unraveling the Physiological Roles of the Cyanobacterium Geitlerinema sp. BBD and Other Black Band Disease Community Members through Genomic Analysis of a Mixed Culture. *PLoS One* **11**, e0157953 (2016).

14. Myers JL, Richardson LL. Adaptation of cyanobacteria to the sulfide-rich microenvironment of black band disease of coral. *FEMS Microbiol Ecol* **67**, 242–251 (2009).

15. Antonaru LA, Nurnberg DJ. Role of PatS and cell type on the heterocyst spacing pattern in a filamentous branching cyanobacterium. *FEMS Microbiol Lett* **364**, (2017).

16. Livingstone D, Pentecost A, Whitton BA. Diel variations in nitrogen and carbon dioxide fixation by the blue-green alga Rivuluria in an upland stream. *Phycologia* **23**, 125–133 (1984).

17. Najeeb S, Khan RAA, Deng X, Wu C. Drivers and consequences of degradation in tropical reef island ecosystems: strategies for restoration and conservation. *Frontiers in Marine Science* **12**, (2025).

18. Garrett P, Ducklow H. Coral disease in Bermuda. *Nature* **253**, 349–350 (1975).

19. Klaus JS, Janse I, Fouke BW. Coral Black Band Disease Microbial Communities and Genotypic Variability of the Dominant Cyanobacteria (CD1C11). *Bulletin of Marine Science* **87**, 795–821 (2011).

20. Huang T, Grobbelaar B. The circadian clock in the prokaryote Synechococcus RF4 *Microbiology* **141**, 535–540 (1995).

21. Spiller H, Shanmugam K. Physiological Conditions for Nitrogen Fixation in a Unicellular Marine Cyanobacterium, Synechococcus sp. Strain SF1. *Journal of Bacteriology* **169**, 5379–5384 (1987).

22. Hadaidi G, Ziegler M, Shore-Maggio A, Jensen T, Aeby G, Voolstra CR. Ecological and molecular characterization of a coral black band disease outbreak in the Red Sea during a bleaching event. *PeerJ* **6**, e5169 (2018).

23. Sunagawa S*, et al.* Bacterial diversity and White Plague Disease-associated community changes in the Caribbean coral Montastraea faveolata. *ISME J* **3**, 512–521 (2009).

24. Arotsker L, Kramarsky-Winter E, Ben-Dov E, Siboni N, Kushmaro A. Changes in the bacterial community associated with black band disease in a Red Sea coral, Favia sp., in relation to disease phases. *Dis Aquat Organ* **116**, 47–58 (2015).

25. Berrendero E*, et al.* Nitrogen fixation in a non-heterocystous cyanobacterial mat from a mountain river. *Sci Rep* **6**, 30920 (2016).

26. Ruetzler K, SANTAVY D. The Black Band Disease of Atlantic Reef Corals. *PSZNI Marine Ecology* **4**, 301–319 (1983).

27. Sekar R, Kaczmarsky LT, Richardson LL. Effect of freezing on PCR amplification of 16S rRNA genes from microbes associated with black band disease of corals. *Appl Environ Microbiol* **75**, 2581–2584 (2009).

28. Sajid S*, et al.* Increased sulfate-reducing bacteria can drive microbial dysbiosis in bleached corals. *J Appl Microbiol* **136**, (2025).

29. Barneah O, Ben-Dov E, Kramarsky-Winter E, Kushmaro A. Characterization of black band disease in Red Sea stony corals. *Environ Microbiol* **9**, 1995–2006 (2007).

30. Viehman S, Mills D, Meichel G, Richardson L. Culture and identification of Desulfovibrio spp. from corals infected by black band disease on Dominican and Florida Keys reefs. *DISEASES OF AQUATIC ORGANISMS* **69**, 119–127 (2006).

31. Ben-Dov E, Siboni N, Shapiro OH, Arotsker L, Kushmaro A. Substitution by inosine at the 3'-ultimate and penultimate positions of 16S rRNA gene universal primers. *Microb Ecol* **61**, 1–6 (2011).

32. Sekar R, Kaczmarsky LT, Richardson LL. Microbial community composition of black band disease on the coral host Siderastrea siderea from three regions of the wider Caribbean. *Marine Ecology Progress Series* **362**, 85–98 (2008).

33. Teske A, Salman V. The Family Beggiatoaceae. In: *The Prokaryotes*) (2014).

34. Brock J, Rhiel E, Beutler M, Salman V, Schulz-Vogt HN. Unusual polyphosphate inclusions observed in a marine Beggiatoa strain. *Antonie Van Leeuwenhoek* **101**, 347–357 (2012).

35. Sorokin. Thioflexithrix psekupsensis, description of a filamentous, sulfide-oxidizing bacterium. . *Int J Syst Evol Microbiol* **60**, 1465–1470 (2010).

36. Han Y, Perner M. The globally widespread genus Sulfurimonas: versatile energy metabolisms and adaptations to redox clines. *Front Microbiol* **6**, 989 (2015).

37. Arotsker L, Siboni N, Ben-Dov E, Kramarsky-Winter E, Loya Y, Kushmaro A. Vibrio sp. as a potentially important member of the Black Band Disease (BBD) consortium in Favia sp. corals. *FEMS Microbiol Ecol* **70**, 515–524 (2009).

38. Bhedi CD*, et al.* Elevated temperature enhances short- to medium-chain acyl homoserine lactone production by black band disease-associated vibrios. *FEMS Microbiol Ecol* **93**, (2017).

39. Sussman M, Willis BL, Victor S, Bourne DG. Coral pathogens identified for White Syndrome (WS) epizootics in the Indo-Pacific. *PLoS One* **3**, e2393 (2008).

40. Zimmer BL*, et al.* Quorum sensing signal production and microbial interactions in a polymicrobial disease of corals and the coral surface mucopolysaccharide layer. *PLoS One* **9**, e108541 (2014).

41. Johan O*, et al.* Microbial community of black band disease on infection, healthy, and dead part of scleractinian montipora sp. colony at seribu islands, indonesia. *Indonesian Aquaculture Journal*, (2014).

42. Agung M, Maqbul I, Astuty S, Mulyani Y. Bacterial community composition among coral diseases in Biawak Island using denaturing gradient gel electrophoresis. *Research Journal of Chemistry and Environment* **24**, (2020).

43. Roder C*, et al.* Bacterial profiling of White Plague Disease in a comparative coral species framework. *ISME J* **8**, 31–39 (2014).

44. Miller AW, Richardson LL. A meta-analysis of 16S rRNA gene clone libraries from the polymicrobial black band disease of corals. *FEMS Microbiol Ecol* **75**, 231–241 (2011).

45. Wada N*, et al.* Microbial mat compositions and localization patterns explain the virulence of black band disease in corals. *NPJ Biofilms Microbiomes* **9**, 15 (2023).

46. Sekar R, Mills DK, Remily ER, Voss JD, Richardson LL. Microbial communities in the surface mucopolysaccharide layer and the black band microbial mat of black band-diseased Siderastrea siderea. *Appl Environ Microbiol* **72**, 5963–5973 (2006).

47. Gantar M, Kaczmarsky LT, Stanic D, Miller AW, Richardson LL. Antibacterial activity of marine and black band disease cyanobacteria against coral-associated bacteria. *Mar Drugs* **9**, 2089–2105 (2011).

48. Meyer JL, Gunasekera SP, Scott RM, Paul VJ, Teplitski M. Microbiome shifts and the inhibition of quorum sensing by Black Band Disease cyanobacteria. *ISME J* **10**, 1204–1216 (2016).

49. Meyer JL, Castellanos-Gell J, Aeby GS, Hase CC, Ushijima B, Paul VJ. Microbial Community Shifts Associated With the Ongoing Stony Coral Tissue Loss Disease Outbreak on the Florida Reef Tract. *Front Microbiol* **10**, 2244 (2019).

50. Progress Toward Restoring the Everglades: The Eighth Biennial Review - 2020. Washington, DC: The National Academies Press (2021).
