# Supplementary material for: Breakdown of microbial networks links nutrient stress and reef coral disease
Source: Nat Commun. 2026 May 5;17:3821. doi: 10.1038/s41467-026-72175-4 (PMC13144614; doi:10.1038/s41467-026-72175-4)
Supplement: Supplementary file 2 — Description of Additional Supplementary Information [file 41467_2026_72175_MOESM2_ESM.pdf]

Description of Additional Supplementary Information

Title: Supplementary Data 1

Description: Primary research papers identified through a Web of Science search between 2000 and 2023 (n=141).

Title: Supplementary Data 2

Description: Statistical analysis of alpha diversity metrics and statistical analysis of Beta-diversity.

Title: Supplementary Data 3

Description: Statistical analysis of network properties.

Title: Supplementary Data 4

Description: Compilation of temperature and nutrient data retrieved from NOAA coral watch and Bio-Oracle data bases. Locations are ordered alphabetically by the first author's name of the corresponding reference (Suppl Table 1). NA= No data available
