# Supplementary material for: Breakdown of microbial networks links nutrient stress and reef coral disease
Source: Nat Commun. 2026 May 5;17:3821. doi: 10.1038/s41467-026-72175-4 (PMC13144614; doi:10.1038/s41467-026-72175-4)
Supplement: Supplementary file 7 — Reporting Summary [file 41467_2026_72175_MOESM7_ESM.pdf]

nature portfolio

Corresponding author(s): Cecilia D'Angelo

Last updated by author(s): Mar 15, 2026

Reporting Summary

Nature Portfolio wishes to improve the reproducibility of the work that we publish. This form provides structure for consistency and transparency in reporting. For further information on Nature Portfolio policies, see our Editorial Policies and the Editorial Policy Checklist.

Statistics

For all statistical analyses, confirm that the following items are present in the figure legend, table legend, main text, or Methods section.

n/a	Confirmed	
☐	☒	The exact sample size (*n*) for each experimental group/condition, given as a discrete number and unit of measurement
☐	☒	A statement on whether measurements were taken from distinct samples or whether the same sample was measured repeatedly
☐	☒	The statistical test(s) used AND whether they are one- or two-sided<br>*Only common tests should be described solely by name; describe more complex techniques in the Methods section.*
☒	☐	A description of all covariates tested
☒	☐	A description of any assumptions or corrections, such as tests of normality and adjustment for multiple comparisons
☐	☒	A full description of the statistical parameters including central tendency (e.g. means) or other basic estimates (e.g. regression coefficient) AND variation (e.g. standard deviation) or associated estimates of uncertainty (e.g. confidence intervals)
☐	☒	For null hypothesis testing, the test statistic (e.g. *F*, *t*, *r*) with confidence intervals, effect sizes, degrees of freedom and *P* value noted<br>*Give P values as exact values whenever suitable.*
☒	☐	For Bayesian analysis, information on the choice of priors and Markov chain Monte Carlo settings
☒	☐	For hierarchical and complex designs, identification of the appropriate level for tests and full reporting of outcomes
☒	☐	Estimates of effect sizes (e.g. Cohen's *d*, Pearson's *r*), indicating how they were calculated

Our web collection on statistics for biologists contains articles on many of the points above.

Software and code

Policy information about availability of computer code

Data collection	Web of Science (with search parameter: including "black band disease" either the title or abstract between 2000 and 2023) was used for the literature search.<br>QGIS (v3.4) was used to collate and map latitude information of sites of disease reports.<br>The SILVA SSU r138 (version 2, 2024) database was used for identification of microbial taxonomy.<br>Daily global 5km satellite coral bleaching heat stress degree heating week data was obtained from NOAA Coral Reef Watch (version 3.1, released August 1, 2018).<br>Global nitrate and phosphate surface seawater concentrations obtained from Bio-ORACLE v3.0.<br>Water flow discharge data were obtained from the US Geological Survey's (USGS) National Water Information System (NWIS). We used data from the gaging station located at Shark River Below Gunboat Island near Flamingo, Florida (USGS site no. 252230081021300) to quantify water flux from the Everglades.
Data analysis	The area of tissue loss was calculated using the polygon tool in ImageJ (version 1.53).<br>All 16s rRNA amplicon sequencing data was processed using R version 4.4.2 and RStudio 2025.05.1+513.<br>For sequence analyses and identification of microbiome compositions we used the DADA2 pipeline, and the DECIPHER and ANCOM-BC2 packages.<br>Amplicon sequences were aligned using the Multiple Alignment using Fast Fourier Transform (MAFFT) alignment program and a maximum likelihood phylogenetic tree was estimated with IG-TREE (v3.0).

For manuscripts utilizing custom algorithms or software that are central to the research but not yet described in published literature, software must be made available to editors and reviewers. We strongly encourage code deposition in a community repository (e.g. GitHub). See the Nature Portfolio guidelines for submitting code & software for further information.

Data

Policy information about availability of data

All manuscripts must include a data availability statement. This statement should provide the following information, where applicable:

- Accession codes, unique identifiers, or web links for publicly available datasets
- A description of any restrictions on data availability
- For clinical datasets or third party data, please ensure that the statement adheres to our policy

The complete list of primary research papers utilised in this study; output details of statistical analyses; and compilation of temperature and nutrient data retrieved from open access data bases are provided in Supplementary Data 1-4. 16S rRNA sequencing count tables and taxonomy, Fv/Fm, and tissue loss measurements data supporting the findings of this study are available via the PURE repository at the University of Southampton UK, subject to standard CC-BY license terms, and can be accessed through the link: https://doi.org/10.5258/SOTON/D3751. Raw 16S rRNA amplicon sequence data has been deposited in the NCBI Sequence Read Archive (SRA) with BioProject ID PRJNA1436489. Background land maps were generated using free vector and raster map data @ naturalearthdata.com.

Research involving human participants, their data, or biological material

Policy information about studies with human participants or human data. See also policy information about sex, gender (identity/presentation), and sexual orientation and race, ethnicity and racism.

Reporting on sex and gender	This research does not involve human participants, their data, or biological material.
Reporting on race, ethnicity, or other socially relevant groupings	This research does not involve human participants, their data, or biological material.
Population characteristics	This research does not involve human participants, their data, or biological material.
Recruitment	This research does not involve human participants, their data, or biological material.
Ethics oversight	This research does not involve human participants, their data, or biological material.

Note that full information on the approval of the study protocol must also be provided in the manuscript.

Field-specific reporting

Please select the one below that is the best fit for your research. If you are not sure, read the appropriate sections before making your selection.

☐ Life sciences ☐ Behavioural & social sciences ☒ Ecological, evolutionary & environmental sciences

For a reference copy of the document with all sections, see nature.com/documents/nr-reporting-summary-flat.pdf

Ecological, evolutionary & environmental sciences study design

All studies must disclose on these points even when the disclosure is negative.

Study description	Coral colonies were cultured and propagated by fragmentation to produce genetically identical replicate fragments or ramets in the experimental mesocosm of The Coral Reef Laboratory, University of Southampton. Coral animal husbandry was performed in compliance with the relevant ethical regulations at the University of Southampton. Coral fragments were allowed to recover for >14 days in optimal growing conditions before random distribution into experimental systems. All coral culture experiments were conducted across three experimental systems, each with a defined seawater N:P stoichiometry.
Research sample	The coral Turbinaria reniformis was studied under controlled laboratory conditions. T. reniformis is not only a well-established experimental model, but also a member of a genus known to be susceptible to BBD in natural reef environments, hence well suited for such disease studies.
Sampling strategy	Three replicate coral fragments were randomly selected from each experimental treatment and sampled for prokaryotic community analysis in healthy tissue areas after at least 7 weeks of treatment on days 50 (n = 9) and 73 (n = 9). Microbial lesion samples (n = 8) were collected on day 60 from HN:LP and LN:HP conditions. For comparison, samples from the remaining healthy tissue of the same colonies were also collected on day 60 from LN:HP (n=1) and HN:LP (n=3). Replicate seawater samples were collected at each sampling timepoint (days 50, 60 and 73; n = 12).
Data collection	Photosystem II (PSII) maximum quantum yield (Fv/Fm) was measured after ≥10h of dark acclimation using a submersible pulse-amplitude modulated fluorometer (Diving-PAM) (Walz).<br>The extent of tissue loss was quantified using photos of coral fragments taken from above. The area of tissue loss was calculated using the polygon tool in ImageJ (version 1.53).<br>16S rRNA gene amplicons and sequencing using the Illumina MiSeq sequencer at the Environmental Sequencing Facility,at the SOES University of Southampton.<br>A literature search was conducted using Web of Science. A search for research papers published in the 24-year period between 2000 and 2023 including "black band disease" in either the title or abstract was conducted. Each paper was downloaded and reviewed individually. Non-relevant papers were not considered further.

	The NOAA Coral Reef Watch (CRW) daily 5-km satellite coral bleaching Degree Heating Week (DHW) product (version 3.1, released August 1, 2018) was used to reconstruct the thermal history of reported BBD events. The Degree Heating Weeks (DHW) data show the accumulated heating levels for a specific location with 5km resolution. We used DHW data for the 365 days prior to a BBD event as metric for anomalously high temperatures experienced by each BBD incidence point.<br>Global N:P seawater stoichiometry was calculated from nitrate and phosphate surface seawater (0 to 0.49 meters depth) concentrations obtained from Bio-ORACLE v3.052, 105 using QGIS and the prevailing N:P conditions were extracted for each BBD incidence point<br>Water flow discharge data were obtained from the US Geological Survey's (USGS) National Water Information System (NWIS). We used data from the gaging station located at Shark River Below Gunboat Island near Flamingo, Florida (USGS site no. 252230081021300) to quantify water flux from the Everglades. We used 20 years of daily discharge data from this location to calculate the long-term monthly mean water flow.
Timing and spatial scale	Coral fragments were allowed to recover for >14 days after fragmentation in optimal growing conditions before random distribution into experimental system.<br>After recovery from fragmentation, replicate coral fragments for T. reniformis (n = 18 fragments) were cultured under the three different seawater N:P stoichiometry systems for >7 weeks.<br>The literature search, conducted using Web of Science, included a search for research papers published in the 24-year period between 2000 and 2023.<br>We used DHW data for the 365 days prior to a BBD event as metric for anomalously high temperatures experienced by each BBD incidence point.<br>Global N:P seawater stoichiometry was calculated from nitrate and phosphate surface seawater (0 to 0.49 meters depth) concentrations, the prevailing N:P conditions were extracted for each BBD incidence point<br>We used data from the gaging station located at Shark River Below Gunboat Island near Flamingo, Florida (USGS site no. 252230081021300) to quantify water flux from the Everglades. We used 20 years of daily discharge data from this location to calculate the long-term monthly mean water flow.
Data exclusions	16S sequence reads were truncated to remove primer sequences and low-quality bases at the 3' end. Reads containing N values or more than two expected errors were removed from the analysis.<br>Chimeric sequence reads and ASVs with a total abundance of <10 across all samples were removed from further analyses.
Reproducibility	Nutrient conditions with high nitrate/high phosphate were used as control, simulating nutrient environments that have previously been described for high nutrient reef environments (Galápagos Islands, Brazilian coast) or in reefs with internal wave-driven upwelling.<br>Photosynthesis measurements in corals exposed to all treatments revealed similar values as those previously published experiments.
Randomization	Randomised design was used to distribute samples across the different treatments.
Blinding	The nature of the physiological studies and sampling strategies of disease areas in this work requires the precise identification of samples.

Did the study involve field work? ☐ Yes ☒ No

Reporting for specific materials, systems and methods

We require information from authors about some types of materials, experimental systems and methods used in many studies. Here, indicate whether each material, system or method listed is relevant to your study. If you are not sure if a list item applies to your research, read the appropriate section before selecting a response.

Materials & experimental systems

n/a	Involved in the study	
☒	☐	Antibodies
☒	☐	Eukaryotic cell lines
☒	☐	Palaeontology and archaeology
☐	☒	Animals and other organisms
☒	☐	Clinical data
☒	☐	Dual use research of concern
☒	☐	Plants

Methods

n/a	Involved in the study	
☒	☐	ChIP-seq
☒	☐	Flow cytometry
☒	☐	MRI-based neuroimaging

Animals and other research organisms

Policy information about studies involving animals; ARRIVE guidelines recommended for reporting animal research, and Sex and Gender in Research

Laboratory animals	Turbinaria reniformis is a coral (non-vertebrate organism) is grown in our laboratories since 2008. All experimental samples were produced through in-house propagation, accordingly the experimental approach is entirely sustainable.

Wild animals	No wild animals were used in this study.
Reporting on sex	Corals are often hermaphrodites, therefore the matter of sex is not applicable in this context.
Field-collected samples	No samples were collected from the field in this study.
Ethics oversight	Ethics are controlled by the Ethics and Research Governance ONLINE, hosted by the University of Southampton.

Note that full information on the approval of the study protocol must also be provided in the manuscript.

Plants

Seed stocks	Seed stocks were not used in this study.
Novel plant genotypes	No novel plant genotypes were used or generated int his study.
Authentication	No novel plant genotypes were used or generated int his study.
